# Evaluation of the Sysmex XQ‐320 three‐part differential haematology analyser and its flagging capabilities

**DOI:** 10.1002/jcla.25017

**Published:** 2024-02-23

**Authors:** Christine Coutaz, Adrien Mamin, Konstantinos Mintzas

**Affiliations:** ^1^ General Haematology Laboratory Centre Hospitalier Universitaire Vaudois (CHUV) Lausanne Switzerland; ^2^ Sysmex Europe SE Norderstedt Germany

**Keywords:** analytical performance, correlation, flagging, haematology analyser, three‐part differential

## Abstract

**Background:**

Three‐part differential (3PD) haematology analysers offer a quick, easy‐to‐use and economical way to acquire important information about a patient's physiology. In this study, we evaluated a new 3PD analyser, the Sysmex XQ‐320, investigated its comparability with its predecessor (Sysmex XP‐300) and the five‐part differential analyser Sysmex XN‐9000, and explored its flagging potential.

**Methods:**

Analytical performance studies were conducted for repeatability, within‐laboratory precision, between‐day precision, carry‐over and linearity with fresh blood and QC material. Method comparison was performed in 493 samples comparing XQ‐320 with XP‐300, using the XN‐9000 as the gold standard.

**Results:**

The XQ‐320 excelled manufacturer's specifications in the analytical performance studies, except for MXD in within‐laboratory and between‐day precisions using the QC material level 1. The XQ‐320 showed correlation values greater than 0.94 with XN‐9000 for the majority of the 20 reportable parameters (MXD# 0.891, MXD% 0.898 and MCHC 0.849). Improvements over the XP‐300 were observed in WBC in the leucocytopenic range (bias −0.038 vs. −0.097) and PLT (bias 2.568 vs. −7.877, intercept 3.880 vs. −8.845). Concordance between XQ‐320 and XP‐300 was 91.9% for the WBC histogram abnormal distribution flag and 95.3% for the PLT flag. Patterns of increased neutrophils and decreased mixed cells on the XQ‐320 were observed in samples that raised a flag on XN‐9000.

**Conclusion:**

The XQ‐320 showed excellent analytical performance, and very good to excellent correlation with XN‐9000 with improvements over XP‐300. Flagging combined with parameter patterns identified additional suspected abnormal samples, thus making the XQ‐320 an excellent solution for laboratories utilising 3PD analysers.

## Graphical abstract

The Sysmex XQ‐320 three‐part differential analyser showed very good to excellent correlation with the Sysmex XN‐9000 in all reportable parameters, with notable improvements over its predecessor XP‐300 in key areas, such as measuring WBC in the leucocytopenic range, and delivering precise PLT values. The XQ‐320 flagging system provides all relevant information at a glance.

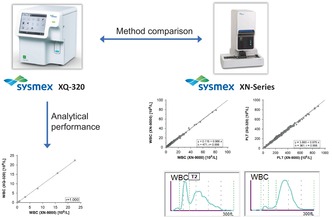

## 1 INTRODUCTION

The complete blood count (CBC) from a haematology analyser offers a comprehensive picture of a person's physiology and is usually the first laboratory examination conducted.^1^ Haematology analysers come in different shapes and forms, from small stand‐alone instruments to large automation lines, offering a wide variety of features depending on the desired usage.^2^ One differentiating factor is the number of white blood cell (WBC) subpopulations that can be identified. A five‐part differential (5PD) analyser can separate lymphocytes, neutrophils, monocytes, eosinophils and basophils, while a three‐part differential (3PD) analyser groups monocytes, eosinophils and basophils in one group usually termed ‘mixed’ population. Analysers can also perform six‐ and seven‐part differentials, by separating immature granulocytes or other immature cell parameters.^3^ In specific settings such as hospital wards or emergency rooms, a small stand‐alone analyser is preferred for obtaining results quicker. This need can be fulfilled by both 5PD and 3PD analysers, but the former usually comes with higher costs per test and the provided results may be harder to interpret than having fewer parameters. The 3PD analysers are usually small stand‐alone instruments, making them easier to use and cost‐efficient to operate, and thus can be found in a diverse range of locations, from laboratories to doctor offices to remote locations.

The Sysmex Corporation has developed a number of 3PD analysers in the last decades. The XP‐300 is being used in the Oncology Service of our hospital since June 2017.^4^ The XQ‐320 is the latest 3PD analyser and is the focus of this study. Both analysers feature 20 diagnostic parameters, but the XQ‐320 comes with several improvements. Wider measuring intervals, higher throughput (70 vs. 60 samples/hour), less aspiration volume (16 vs. 50 μL), faster results and a larger screen giving more details and information at a glance. On the other end of the technology spectrum, the Sysmex XN‐9000 5PD haematology analyser is used in the central laboratory of our hospital. This analyser has the additional capability of performing six‐part differential analysis, where immature granulocytes are measured independently from neutrophils.^5^

In this study, we sought to evaluate the analytical performance of the XQ‐320, perform method comparison with the XP‐300 using the XN‐9000 as the gold standard method, and explore the flagging capabilities of this new 3PD system.

## 2 MATERIALS AND METHODS

The study was conducted in the General Haematology laboratory of the Centre Hospitalier Universitaire Vaudois (CHUV) in Lausanne, Switzerland. The laboratory is equipped with a Sysmex XN‐9000 haematology analyser. For the duration of the study, a Sysmex XQ‐320 and a Sysmex XP‐300 were additionally installed. All analysers (Sysmex Corporation, Kobe, Japan) were calibrated per manufacturer specifications, and quality control materials were ran daily.

### 2.1 Measurement principles

The Sysmex XN‐9000 haematology analyser is a six‐part differential analyser utilising fluorescence flow cytometry to differentiate the six populations of the differential (lymphocytes, monocytes, neutrophils, basophils, eosinophils, and immature granulocytes). The Sysmex XQ‐320 and Sysmex XP‐300 haematology analysers are 3PD analysers that utilise a DC detection method for measuring white blood cells and dividing them into three subgroups based on their size (lymphocytes, mixed cells and neutrophils).

### 2.2 Parameters and flagging

The 20 reportable parameters of the XQ‐320 were evaluated among the three systems (Table 1). For the purposes of this study, the immature granulocytes were added in the neutrophil measurement on the XN‐9000 (representing the 5PD setting of the analyser). Additionally, a ‘MXD’ parameter was calculated for the XN‐9000 by adding the monocyte, basophil and eosinophil measurements in each sample.

**TABLE 1:** Parameters from Sysmex XQ‐320, XP‐300 and XN‐9000 analysers investigated in this study.

			XQ‐320, XP‐300	XN‐9000
White blood cell‐related parameters				
WBC	White blood cell count	10^9^/L	O	O
NEUT#/%^a^	Neutrophil count and percent	10^9^/L, %	O	O
LYMPH#/%	Lymphocyte count and percent	10^9^/L, %	O	O
MONO#/%	Monocyte count and percent	10^9^/L, %		O
BASO#/%	Basophil count and percent	10^9^/L, %		O
EO#/%	Eosinophil count and percent	10^9^/L, %		O
IG#/%	Immature granulocyte count and percent	10^9^/L, %		O
MXD#/%^b^	Mixed cell absolute count and percent	10^9^/L, %	O	
Red blood cell‐related parameters				
RBC	Red blood cell count	10^12^/L	O	O
HGB	Haemoglobin concentration	g/L	O	O
HCT	Haematocrit	%	O	O
MCV	Mean corpuscular volume	fL	O	O
MCH	Mean corpuscular haemoglobin	pg	O	O
MCHC	Mean corpuscular haemoglobin concentration	g/L	O	O
RDW‐SD	Red cell distribution width—standard deviation	fL	O	O
RDW‐CV	Red cell distribution width—coefficient of variation	%	O	O
Platelet‐related parameters				
PLT	Platelet count	10^9^/L	O	O
PDW	Platelet distribution width	fL	O	O
MPV	Mean platelet volume	fL	O	O
P‐LCR	Platelet large cell ratio	%	O	O
PCT	Plateletcrit	%	O	O

^a^ For comparison studies, NEUT#/% on XN‐9000 derived from the 5‐part DIFF setting; IG#/% were included in NEUT.

^b^ For comparison studies, MXD#/% on XN‐9000 is calculated as MONO+BASO+EO.

The XN‐9000 alerts the user for abnormalities in the samples with suspect flags for the possible presence of pathologic cells, and with abnormal flags for parameter values outside the normal range and/or abnormal histograms or scattergrams. The XQ‐320 carries the abnormal flags of the XN‐9000 and some of the suspect ones (there are no suspect flags for WBC abnormalities). Additionally, distribution flags indicate the specific abnormality of the histogram that leads to the abnormal distribution. The XP‐300 has only the distribution flags for the WBC, RBC and PLT histograms.

### 2.3 Analytical performance studies

The assessment of the XQ‐320 analytical performance was conducted with protocols adapted from the ‘ICSH guidelines for the evaluation of blood cell analysers’^6^ and the ‘CLSI EP‐15‐A3’.^7^ Results were compared against the manufacturer specifications stated in the instructions for use (IFU) and where applicable with the biological variation (BV) from the EFLM database.^8^ Due to the lack of BV data for the MXD parameter, the data from monocytes were used.

#### 2.3.1 Repeatability (within‐run precision)

During the first day of the study, two samples with the following criteria were randomly selected from the XN‐9000 routine workflow: a sample with several parameters outside the normal range (positive), and a sample with all parameters within normal range (negative). These samples were measured 10 consecutive times on the XQ‐320. The coefficient of variation (CV%) was calculated from the mean and standard deviation (SD) values for the 20 reportable parameters of XQ‐320.

#### 2.3.2 Within‐laboratory precision

All three levels of the EIGHTCHECK‐3WP QC material (level 1 and 3—lot 2253, level 2—lot 2254) were measured on the XQ‐320 in duplicates in the morning and in the afternoon for 5 days. Two‐way ANOVA (first factor: different days; second factor: different time within each day) was used to calculate statistical variations between measurements. The mean and SD for each parameter were used to calculate the CV% values for the 20 reportable parameters of XQ‐320.

#### 2.3.3 Between‐day precision

All three levels of the EIGHTCHECK‐3WP QC material (level 1 and 3—lot 2253, level 2—lot 2254) were measured once daily for 21 days on the XQ‐320. The CV% values for the 20 reportable parameters of XQ‐320 were calculated from the mean and SD values.

#### 2.3.4 Carry‐over

The carry‐over evaluation was conducted with both fresh blood and QC material. Four fresh blood samples with high values (above the normal range) and another four with low values for (a) WBC, (b) RBC, (c) HGB and HCT, and (d) PLT were identified on the XN‐9000 during the routine laboratory work time. The EIGHTCHECK‐3WP level 3 (representing higher cell count—lot 2253) and the CELLPACK diluent were additionally used. For each parameter, the sample with the high value was measured three consecutive times on the XQ‐320, followed by the measurement of the sample with the low value for three consecutive times. The carry‐over rate for each parameter was calculated with the following formula: Carry‐over rate (%) = (Low first measurement − Low third measurement)/(High third measurement − Low third measurement) × 100.

#### 2.3.5 Linearity

Fresh blood samples with high values (above the normal range) for WBC, RBC, HGB, HCT and PLT were identified on the XN‐9000 during the routine laboratory work time. Each sample was serially diluted seven times with CELLPACK, until the limit of quantification was reached. The initial sample and the seven dilutions were measured five times on XN‐9000 (theoretic values) and five times on XQ‐320 (result values). The mean values were plotted in a regression graph and the correlation coefficient was calculated.

### 2.4 Method comparison

A part of the routine peripheral blood samples arriving in the General Haematology laboratory of the Centre Hospitalier Universitaire Vaudois (CHUV) in Lausanne, Switzerland during a three‐week period from 28/11/2022 to 16/12/2022 were considered for the study. Blood samples were collected in S‐Monovette K_3_EDTA tubes (Sarstedt AG & Co. KG, Nümbrecht, Germany) and were measured per laboratory protocol on the Sysmex XN‐9000 analyser. While the ICSH guidelines recommend a minimum sample size of 250–300 for analyser evaluations, we opted for 500 samples to ensure greater precision in our estimates and sufficient power to detect clinically relevant differences between the two analysers.^6^ Approximately 30 samples each day with sufficient residual volume from the routing analysis on the XN‐9000 were additionally measured on the Sysmex XQ‐320 and Sysmex XP‐300 analysers within 6 h of collection. Half of the study cohort consisted of randomly selected samples. For the rest, priority was given in the following common flags (as identified on XN‐9000): ‘WBC Abn Scattergram’, ‘Blasts/Abn Lympho?’, ‘IG Present’, ‘NRBC Present’, ‘Monocytosis’, ‘Eosinophilia’, ‘Basophilia’, ‘RBC Abn Distribution’, ‘Fragments?’, ‘RBC agglutination?’, ‘Anaemia’, ‘Anisocytosis’, ‘PLT Abn Distribution’, ‘Giant Platelet?’. Finally, a total of 493 samples with measurements in all three analysers were collected and analysed.

### 2.5 Data analysis

The data from the three analysers were collected and organised in a single master table using Microsoft Excel (Microsoft Corporation, Redmond, United States). The MedCalc statistical software (MedCalc Software, Ostend, Belgium) was used for subsequent analyses. Mean, SD and coefficient of variation (CV%) were calculated for each parameter in repeatability, within‐laboratory precision and between‐day precision studies. For within‐laboratory precision, two‐way ANOVA was used to calculate statistical variations between measurements. Correlation coefficient, a linear regression graph, the regression equation and a Bland–Altman analysis were performed for linearity and method comparison analyses. Correlation coefficients between 0.850 and 0.900 were considered ‘very good’; higher than 0.900 were considered ‘excellent’. Flag concordance was calculated as the ratio of the number of samples with flags in both XQ‐320 and XP‐300 to the number of samples without flag in both analysers, expressed in percent. A *t*‐test was performed to compare the mean parameter values between subgroups of samples with or without selected flags. A *p* value less than 0.05 was considered statistically significant.

### 2.6 Ethical considerations

The current study was classified as a performance evaluation study that did not require clinical information, and no additional blood draw was required; thus, ethical approval was not requested.

## 3 RESULTS

### 3.1 Analytical performance

#### 3.1.1 Repeatability

The repeatability (within‐run precision) CV (%) values for WBC were 1.51% (positive sample) and 1.29% (negative sample); for the differential parameters, they ranged from 1.79% (NEUT% for the negative sample) to 11.28% (MXD# for the negative sample); for RBC and the RBC‐related parameters, CV values remained below 1.32%; for the PLT‐related parameters, they ranged from 1.03% (MPV for the negative sample) to 4.62% (PDW for the negative sample). Detailed results are shown in Table 2. All repeatability values for both samples performed better than the specifications of the XQ analyser, as stated in the instructions for use (IFU), and were lower than the respective biological variation (except MCH).^8^

**TABLE 2:** Repeatability (within‐run precision) study.

	Positive sample		Negative sample		IFU	BV
	CV (%)	Mean	CV (%)	Mean	CV (%)	CV (%)
WBC	1.51	10.81 × 10^9^/L	1.29	5.62 × 10^9^/L	≤3.5	8.3
NEUT#	7.77	2.93 × 10^9^/L	1.87	3.18 × 10^9^/L	≤15.0	14.0
LYMPH#	2.37	5.85 × 10^9^/L	3.19	2.14 × 10^9^/L	≤15.0	10.8
MXD#	9.00	2.02 × 10^9^/L	11.28	0.31 × 10^9^/L	≤30.0	13.3
NEUT%	7.46	27.13%	1.79	56.47%	≤15.0	14.0
LYMPH%	2.00	54.17%	2.83	38.01%	≤15.0	10.8
MXD%	8.90	18.70%	10.02	5.52%	≤30.0	13.3
RBC	1.10	2.68 × 10^12^/L	0.67	4.43 × 10^12^/L	≤2.0	2.8
HGB	0.40	78.9 g/L	0.50	134.0 g/L	≤1.5	2.7
HCT	1.08	24.56%	0.84	39.74%	≤2.0	2.8
MCV	0.48	91.59 fL	0.40	89.74 fL	≤2.0	0.8
MCH	1.02	29.42 pg	1.06	30.26 pg	≤2.0	0.7
MCHC	1.00	321.4 g/L	1.22	337.5 g/L	≤2.0	1.0
RDW‐SD	1.32	58.65 fL	1.31	41.17 fL	≤4.0	1.7
RDW‐CV	1.16	19.56%	1.19	12.06%	≤4.0	—
PLT	N/A	51.50 × 10^9^/L	4.95	242.1 × 10^9^/L	≤6.0	7.3
PDW	N/A	14.38 fL	4.62	11.56 fL	≤12.0	3.8
MPV	N/A	9.65 fL	1.03	9.98 fL	≤5.0	2.3
P‐LCR	N/A	27.73%	2.63	25.65%	≤20.0	6.8
PCT	N/A	0.05%	4.81	0.24%	≤9.0	6.4

Abbreviations: BV, biological variation; IFU, instruction for use specifications; N/A, Specifications for PLT‐related parameters require PLT >100 × 10^9^/L.

#### 3.1.2 Within‐laboratory precision

The within‐laboratory precision CV (%) values for the WBC‐related parameters ranged from 1.18% (WBC in QC material level 3) to 33.87% (MXD% in QC material level 1); for the RBC‐related parameters, CV values ranged from 0.36% (MCV in QC material level 3) to 2.68% (RDW‐CV in QC material level 1); for the PLT‐related parameters, CV values ranged from 0.77% (MPV in QC material level 3) to 10.65% (P‐LCR in QC material level 1). Detailed results are shown in Table 3. Except for MXD# (33.33%) and MXD% (33.87%) in QC material level 3, all within‐laboratory precision values for all three QC levels performed better than the specifications of the XQ analyser, as stated in the IFU. The majority of precisions remained lower than the respective biological variation.

**TABLE 3:** Within‐laboratory precision study.

	QC material level 1		QC material level 2		QC material level 3		BV
	Study CV (%)	IFU CV (%)	Study CV (%)	IFU CV (%)	Study CV (%)	IFU CV (%)	CV (%)
WBC	1.71	≤12	2.04	≤7	1.18	≤7	8.3
NEUT#	4.08	≤20	2.16	≤10	1.43	≤10	14.0
LYMPH#	4.05	≤25	3.33	≤15	1.50	≤15	10.8
MXD#	33.33	≤30	7.69	≤25	5.14	≤25	13.3
NEUT%	3.96	≤15	1.44	≤7	1.42	≤7	14.0
LYMPH%	3.72	≤20	1.99	≤15	1.26	≤15	10.8
MXD%	33.87	≤30	7.45	≤25	4.41	≤25	13.3
RBC	1.82	≤5	1.03	≤4	0.55	≤4	2.8
HGB	0.89	≤5	0.69	≤3	0.84	≤3.5	2.7
HCT	1.81	≤8.5	1.09	≤7.5	0.68	≤7.5	2.8
MCV	0.61	≤6	0.43	≤5	0.36	≤5	0.8
MCH	1.58	≤6	1.35	≤5.5	0.96	≤5.5	0.7
MCHC	1.85	≤8	1.43	≤7	1.07	≤7	1.0
RDW‐SD	1.81	≤20	1.65	≤15	1.35	≤15	1.7
RDW‐CV	2.68	≤20	2.54	≤15	2.59	≤15	—
PLT	5.19	≤30	2.88	≤15	2.19	≤13	7.3
PDW	4.97	≤20	4.08	≤10	2.80	≤10	3.8
MPV	1.72	≤10	1.02	≤8	0.77	≤8	2.3
P‐LCR	10.65	≤60	5.34	≤50	5.17	≤50	6.8
PCT	6.42	≤43	2.96	≤24	3.05	≤20	6.4

Abbreviations: BV, biological variation; IFU, instruction for use specifications.

#### 3.1.3 Between‐day precision

The between‐day precision CV (%) values for the WBC‐related parameters ranged from 1.12% (LYMPH% in QC material level 3) to 30.36% (MXD% with QC level 1); for the RBC‐related parameters, CV values ranged from 0.49% (MCV with QC level 3) to 3.36% (RDW‐CV in QC material level 2); for the PLT‐related parameters, they ranged from 0.99% (MPV in QC material level 3) to 14.74% (P‐LCR in QC material level 1). Detailed results are shown in Table 4. With the exception of MXD# (30.25%) and MXD% (30.36%) in the QC level 3, all between‐day precision values for all three QC levels performed better than the specifications of the XQ analyser, as stated in the IFU. The majority of precisions remained lower than the respective biological variation.

**TABLE 4:** Between‐day precision study.

	QC material level 1		QC material level 2		QC material level 3		BV
	Study CV (%)	IFU CV (%)	Study CV (%)	IFU CV (%)	Study CV (%)	IFU CV (%)	CV (%)
WBC	3.41	≤12	1.56	≤7	1.36	≤7	8.3
NEUT#	5.76	≤20	3.01	≤10	2.33	≤10	14.0
LYMPH#	5.97	≤25	2.17	≤15	1.49	≤15	10.8
MXD#	30.25	≤30	9.27	≤25	4.36	≤25	13.3
NEUT%	4.04	≤15	1.95	≤7	1.60	≤7	14.0
LYMPH%	4.15	≤20	1.72	≤15	1.12	≤15	10.8
MXD%	30.36	≤30	9.81	≤25	4.26	≤25	13.3
RBC	2.04	≤5	1.40	≤4	0.95	≤4	2.8
HGB	1.61	≤5	0.92	≤3	1.06	≤3.5	2.7
HCT	2.19	≤8.5	1.51	≤7.5	9.68	≤7.5	2.8
MCV	0.83	≤6	0.65	≤5	0.49	≤5	0.8
MCH	1.22	≤6	1.08	≤5.5	0.81	≤5.5	0.7
MCHC	1.57	≤8	1.24	≤7	0.93	≤7	1.0
RDW‐SD	2.70	≤20	1.83	≤15	1.58	≤15	1.7
RDW‐CV	2.72	≤20	3.36	≤15	3.07	≤15	—
PLT	9.34	≤30	3.58	≤15	2.53	≤13	7.3
PDW	7.76	≤20	4.23	≤10	1.58	≤10	3.8
MPV	2.18	≤10	1.65	≤8	0.99	≤8	2.3
P‐LCR	14.74	≤60	5.60	≤50	4.95	≤50	6.8
PCT	8.83	≤43	4.01	≤4	3.15	≤20	6.4

#### 3.1.4 Carry‐over

The carry‐over rate using fresh blood samples was 0.03% for WBC, 0.68% for RBC, and 0.00% for HGB, HCT and PLT. The carry‐over rate with QC material level 3 (representing a higher cell count) was 0.19% for RBC, 0.22% for HCT, and 0.00% for WBC, HGB and PLT. All carry‐over rate values performed better than the specifications of the XQ analyser, as stated in the IFU (WBC: ≤3.0%, RBC, HGB, HCT: ≤1.5%, PLT: ≤5.0%).

#### 3.1.5 Linearity

**FIGURE 1:**
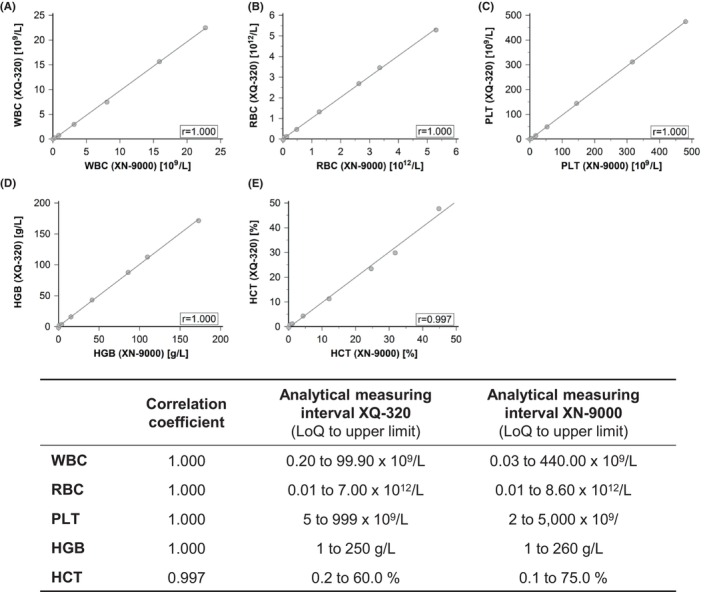
Linearity study. Samples with high values for white blood cell count (WBC), red blood cell count (RBC), haemoglobin concentration (HGB), haematocrit (HCT) and platelet count (PLT) were serially diluted seven times until the limit of quantification was reached. Each dilution was measured five times on XN‐9000 and XQ‐320. The mean values from each replicate were plotted in correlation graphs for WBC (A), RBC (B), PLT (C), HGB (D), and HCT (E). The correlation coefficient (*r*) is shown for each analysis.

The linearity for all tested parameters across a wide range of concentrations was excellent: 1.000 for WBC, RBC, HGB and PLT, and 0.997 for HCT (Figure 1).

### 3.2 Method comparison in 493 samples

#### 3.2.1 WBC‐related parameters

**FIGURE 2:**
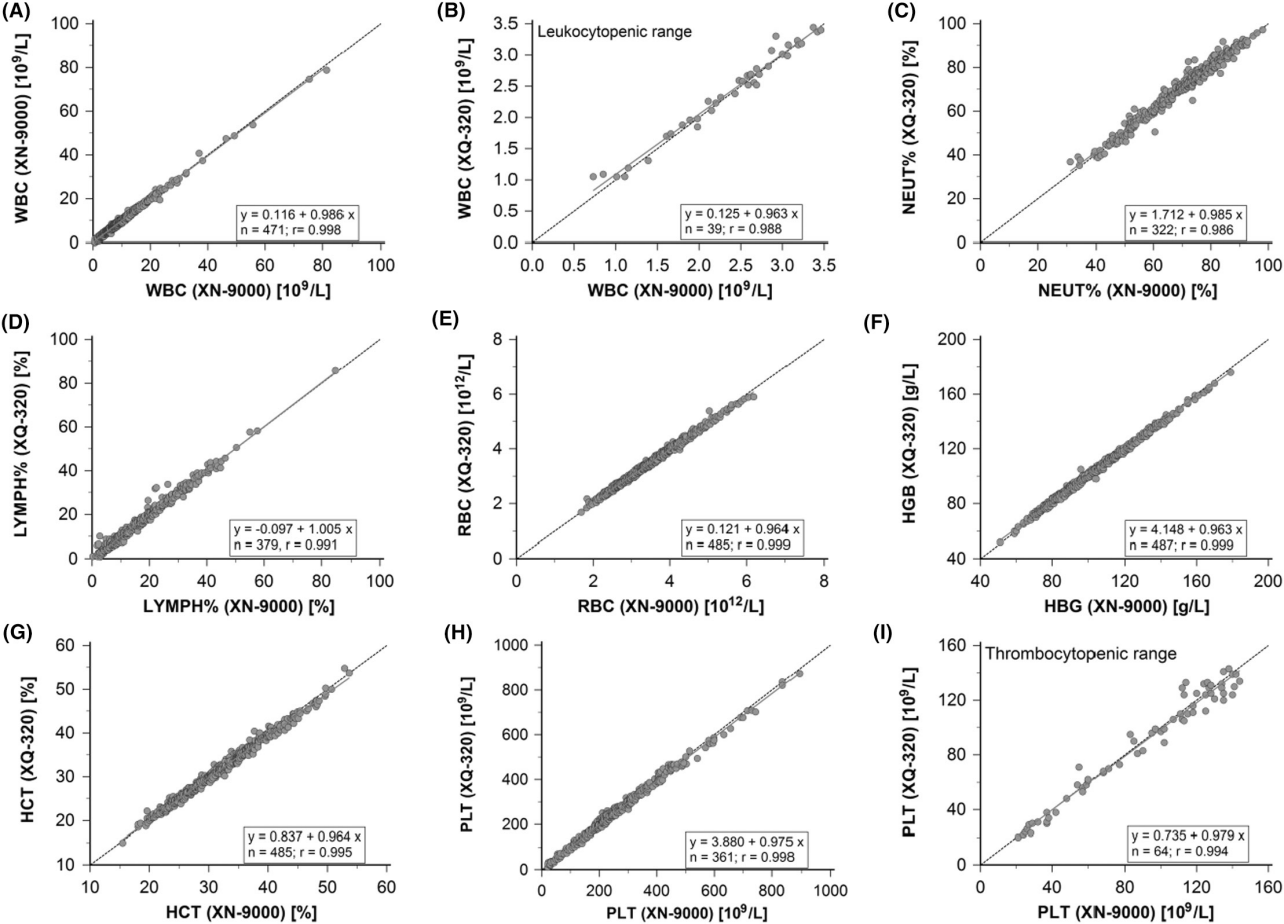
Correlation of selected parameters between XQ‐320 and XN‐9000. Blood samples (*n* = 493) were measured on the Sysmex XQ‐320 and Sysmex XN‐9000 analysers. Correlation graphs with regression line (solid), lines of equality (dashed), regression equation and correlation coefficient (*r*) are shown for white blood cells (WBC) (A), WBC in the leucocytopenic range (<150 × 10^9^/L) (B), neutrophil percent (NEUT%) (C), lymphocyte percent (LYMPH%) (D), red blood cell (RBC) (E), haemoglobin concentration (HBG) (F), haematocrit (HCT) (G), platelet count (PLT) (H), and PLT in the thrombocytopenic range (<150 × 10^9^/L) (I).

**FIGURE 3:**
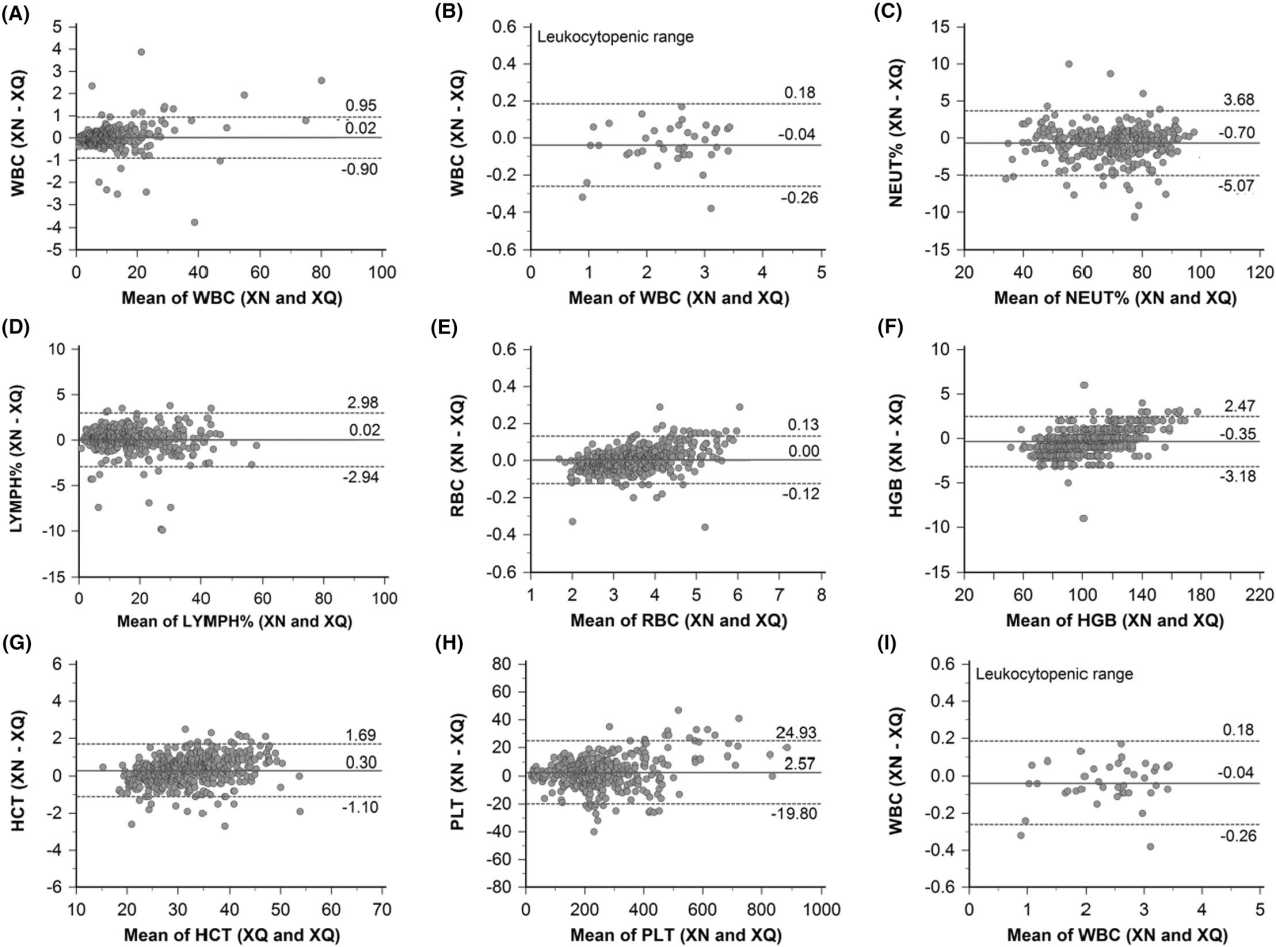
Bland–Altman analysis of selected parameters between XQ‐320 and XN‐9000. Blood samples (*n* = 493) were measured on the Sysmex XQ‐320 and Sysmex XN‐9000 analysers. Bland–Altman plots with bias (difference of means XN—XQ; solid line) and range (1.96SD; dotted lines) are shown for white blood cells (WBC) (A), WBC in the leucocytopenic range (<150 × 10^9^/L) (B), neutrophil percent (NEUT%) (C), lymphocyte percent (LYMPH%) (D), red blood cell (RBC) (E), haemoglobin concentration (HBG) (F), haematocrit (HCT) (G), platelet count (PLT) (H), and PLT in the thrombocytopenic range (<150 × 10^9^/L) (I).

The correlation coefficient (*r*) between XQ‐320 and XN‐9000 for the WBC‐related parameters ranged from very good to excellent; WBC: 0.998, WBC (<3.5 × 10^9^/L): 0.988, NEUT#: 0.998, LYMPH#: 0.953, MXD#: 0.891, NEUT%: 0.986, LYMPH%: 0.991, and MXD%: 0.898. Regression analysis showed that the intercept remained close to zero, and the slope was close to one. No significant bias (XN‐9000 mean—XQ‐320 mean) was observed: WBC: 0.022, WBC (<3.5 × 10^9^/L): −0.038, NEUT#: −0.208, LYMPH#: 0.015, MXD#: 0.060, NEUT%: −0.697, LYMPH%: 0.017, and MXD%: 0.635 (Table 5 and Figures 2 and 3).

**TABLE 5:** Comparison study between XQ‐320 and XN‐9000.

	Sample size	Correlation coefficient	Intercept	Slope	Difference of means (XN‐XQ)
WBC	471	0.998	0.116	0.986	0.022
WBC < 3.5	39	0.988	0.125	0.963	−0.038
NEUT#^a^	322	0.998	0.058	1.001	−0.064
LYMPH#	379	0.953	0.062	0.944	0.015
MXD#^b^	319	0.891	0.068	0.874	0.060
NEUT%^a^	322	0.986	1.712	0.985	−0.697
LYMPH%	379	0.991	‐0.097	1.005	0.017
MXD%^b^	319	0.898	0.352	0.919	0.635
RBC	485	0.999	0.121	0.964	0.004
HGB	487	0.999	4.148	0.963	‐0.353
HCT	485	0.995	0.837	0.964	0.298
MCV	485	0.985	3.305	0.955	0.854
MCH	480	0.983	0.920	0.974	−0.138
MCHC	480	0.849	77.166	0.776	−4.463
RDW‐SD	485	0.941	4.282	0.873	2.309
RDW‐CV	486	0.968	7.757	0.858	−0.353
PLT	361	0.998	3.880	0.975	2.568
PLT < 150	64	0.994	0.735	0.979	1.156
PDW	360	0.894	0.329	0.960	0.154
MPV	360	0.973	1.659	0.811	0.340
P‐LCR	360	0.950	2.030	0.891	1.156
PCT	347	0.994	0.001	0.959	0.010

^a^ For comparison studies, NEUT#/% on XN‐9000 derived from the 5‐part DIFF setting; IG#/% were included in NEUT.

^b^ For comparison studies, MXD#/% on XN‐9000 is calculated as MONO + BASO + EO.

Similar results were observed between XP‐300 and XN‐9000. The correlation coefficient ranged from 0.883 (MXD%) to 0.998 (WBC), with similar slope and intercept values. A higher positive bias for XP‐300 was observed for WBC (0.097) in samples in the leucocytopenic range (<3.5 × 10^9^/L) (Table S1 and Figures S1 and S2).

#### 3.2.2 RBC‐related parameters

The correlation coefficient (*r*) between XQ‐320 and XN‐9000 for the RBC‐related parameters ranged from very good to excellent; RBC: 0.999, HGB: 0.999, HCT: 0.995, MCV: 0.985, MCH: 0.983, MCHC: 0.849, RDW‐SD: 0.941, and RDW‐CV: 0.968. Several parameters had an intercept higher than zero (HGB: 4.148, MCV: 3.305, MCHC: 77.166, and RDW‐SD: 4.282), but the slope remained close to one. MCHC had a positive bias (4.463) and RDW‐SD a negative bias (2.309) on XQ‐320 (Table 5 and Figures 2 and 3).

Similar results were observed between XP‐300 and XN‐9000. The correlation coefficient ranged from 0.902 (MCHC) to 0.999 (RBC and HGB), with HGB, MCV, MCHC and RDW‐SD exhibiting high intercept values. On XP‐300, a positive bias for MCHC was also present (3.768) (Table S1 and Figures S1 and S2).

#### 3.2.3 PLT‐related parameters

The correlation coefficient (*r*) between XQ‐320 and XN‐9000 for the PLT‐related parameters ranged from very good to excellent as well; PLT: 0.998, PLT (<150 × 10^9^/L): 0.994, PDW: 0.894, MPV: 0.973, P‐LCR: 0.950, and PCT: 0.994. A high intercept was observed for the whole range of PLT values (3.880), but was notably lower in the thrombocytopenic range (0.735). A negative bias for PLT was observed on XQ‐320 (2.568) (Table 5 and Figures 2 and 3).

Similar results were observed between XP‐300 and XN‐9000. The correlation coefficient ranged from 0.903 (PDW) to 0.995 (PLT). A more pronounced intercept was observed for PLT (whole range: −8.845, thrombocytopenic range: −4.556), with a much higher bias, albeit positive (7.877) (Table S1 and Figures S1 and S2).

### 3.3 Flagging comparison

#### 3.3.1 Histogram abnormal distribution flags concordance

A comparison of commonly flagged or negative samples in this study cohort showed that XQ‐320 and XP‐300 had a 91.9% agreement (concordance) for ‘WBC Abn Distribution’; XQ‐320 flagged 54 samples, XP‐300 flagged 74, from which 44 were flagged by both systems. When examining only the samples in the leucocytopenic range (<3.5 × 10^9^ WBC/L), concordance remained high at 87%.

Concordance for ‘PLT Abn Distribution’ was even higher at 95.3%; XQ‐320 flagged 60 samples, XP‐300 flagged 59, from which 48 were flagged by both systems. Concordance in the thrombocytopenic range (<150 × 10^9^ PLT/L) was 87.9%. A similar analysis for ‘RBC Abn Distribution’ was not possible, due to the very low number of flagged samples (five samples on each system). Analysis of the different triggers behind ‘WBC Abn Distribution’ and ‘PLT Abn Distribution’ showed no significant differences between XQ‐320 and XP‐300.

#### 3.3.2 Platelet histogram abnormalities

The ‘PLT Abn Distribution’ flag was raised in 90 samples on the XN‐9000, in 60 samples on XQ‐320 and in 59 samples on XP‐300. From the 30 samples that did not trigger a flag on XQ‐320, half had normal platelet count, and half were in the thrombocytopenic range (<150 × 10^9^/L). Detailed analysis of the 15 thrombocytopenic samples, where precise platelet enumeration is more clinically important, showed that the most common feature was a concurrent increase in PDW and P‐LCR in 13 samples. From the remaining two samples, one had dashed‐out values, so this increase was observed in 93% of non‐flagged samples.

#### 3.3.3 Histogram and parameter patterns based on XN‐9000 flags

Since the more extensive technology and flagging system of the XN‐9000 cannot be directly compared to that of any 3PD analyser, we decided to investigate possible 3PD histogram and parameter patterns in abnormal samples, as identified by XN‐9000. For that reason, we compared samples with or without a ‘WBC Abn Distribution’ on the 3PD analysers in subgroups of samples with ‘IG Present’, because we hypothesised that immature granulocytes could have a direct effect on the neutrophil count.

**FIGURE 4:**
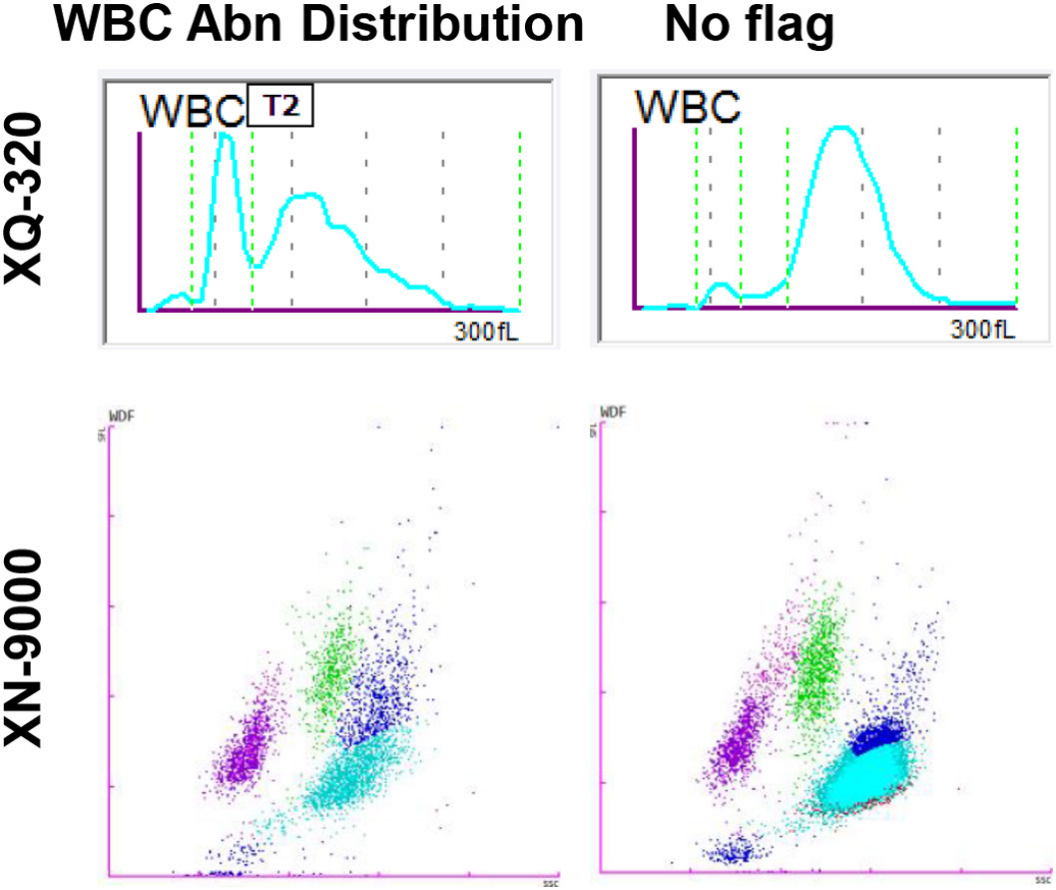
Representative examples of ‘IG Present’ samples. XQ‐320 WBC histograms and XN‐9000 WDF scattergrams from a sample with ‘WBC Abn Distribution’ flag (left panels) and a sample without (right panels).

The ‘IG Present’ flag was triggered in 86 samples. Samples with a ‘WBC Abn Distribution’ on XQ‐320 (*n* = 26), compared to those without (*n* = 60), had increased IG% (12.43 vs. 9.32 × 10^9^/L, *p* = 0.0286), decreased NEUT% but still within normal range (51.58 vs. 65.05%, *p* = 0.0008), decreased NEUT# (3.91 vs. 11.69 × 10^9^/L, *p* = 0.0051), and increased MONO% (16.98 vs. 7.47%, *p* < 0.0001) as measured on the XN‐9000. Examination of representative WBC histograms (from XQ‐320) and WDF scattergrams (from XN‐9000), identified two patterns. A higher IG presence, which on the XN‐9000 leads to a clearer separation from the neutrophil population, affects the WBC histogram on the XQ‐320 where the neutrophil population is not well separated (T2 was present in 16 cases), and an abnormal distribution flag is triggered. In contrast, in those samples with lower IG count, where a clear population separation has not taken place, the neutrophil population is well defined on the histogram without signs of abnormality, and thus the XQ‐320 is not generating an abnormal distribution flag (Figure 4).

## 4 DISCUSSION

3PD analysers have a world‐wide reach and are still the preferable option in selected cases over high‐end 5PD analysers, due to their versatility as light, compact, and easy‐to‐use. Despite the limited WBC classification, they still provide a complete blood profile with all important information for an initial screening directly at a hospital ward, a doctor's office or a satellite laboratory. In this study, our main focus was to evaluate the analytical performance of the new Sysmex XQ‐320 3PD haematology analyser and perform a method comparison with the Sysmex XP‐300, the previous‐generation analyser, using the Sysmex XN‐9000 as the gold standard.

The XQ‐320 excelled in the analytical performance studies compared to the manufacturer specifications and the biological variation, with minimal exceptions. Method comparison for the XP‐300 has been conducted before,^4^ but was repeated here in order to have a direct comparison with the XQ‐320 in our cohort that was enriched with abnormal samples. Very good to excellent correlations were observed for the majority of the parameters for both analysers. Two parameters with lower correlations were MXD and MCHC. MXD are the middle‐sized cells in the WBC histogram of these 3PD analysers and usually have low numbers (MXD# mean of 0.96 × 10^9^/L in our cohort). Small deviations in the lymphocytes (small‐sized cells; mean 1.37 × 10^9^/L) or especially neutrophils (large‐sized cells; mean 6.93 × 10^9^/L) could have a big effect in the determination of MXD, which could explain the lower correlations among the three analysers. The nature of inconsistencies in the MXD measurement have been described in other 3PD analysers, such as the Sysmex pocH‐100i and Sysmex KX‐21N, where the coefficient of determination (R^2^) between the two systems was 0.625 for MXD# and 0.629 for MXD%.^9^ MCHC is a calculated parameter from the ratio of HGB and RBC. Although HGB and RBC had excellent correlations, any slight variation of either one between two analysers could have a greater impact in the HGB/RBC ratio. Suboptimal correlation or insufficient analytical performance has been described for MCHC in previous evaluations of 3PD analysers.^4^, ^9^ Based on our diagnostic workflow, the MCHC value is not as relevant as HGB or RBC (anaemia monitoring) for a first‐line screening of a patient in typical rapid diagnostic situations, such as an oncology ward, thus we do not consider these results as a limitation.

XQ‐320 showed improved performance in the following areas: there was no bias in the WBC on the leucocytopenic range, in contrast to the XP‐300 and we hypothesise that this could be attributed to the broader measuring interval of the XQ‐320 for the WBC (0.2–99.9 × 10^9^/L vs. 1.0–99.9 × 10^9^/L) which makes it more precise in the low range Finally, although the XQ‐320 had an increased intercept and slight negative bias for PLT, the deviations for the XP‐300 were more pronounced. Along with the high intercept for PLT in the thrombocytopenic range, we consider the pattern of the XQ‐320 results as an advantage because there is less risk to misclassify a thrombocytopenic sample as a normal one.

Next, we turned our attention to the flagging systems. In our cohort, enriched with WBC‐ and PLT‐related abnormalities, we observed high concordance between XQ‐320 and XP‐300 for WBC and PLT abnormal histogram distributions. When compared to the XN‐9000, we observed that the 5PD analyser flagged 30 more samples for ‘PLT Abn Distribution’ compared to the XQ‐320. Differences in the underlying technology or algorithms could explain this deviation. Experienced laboratory personnel could identify those samples due to the not ideal appearance of the histogram by using the screen directly on the analyser. For the 15 samples in the thrombocytopenic range, we identified an additional pattern of increased PDW and P‐LCR and we would propose this as an additional rule to be tested and eventually implemented in the software.

Finally, we explored similar patterns on the XQ‐320 for WBC‐related abnormalities, using the highly sensitive and specific XN‐9000 as a reference.^5^, ^10^ A portion of the abnormal samples with ‘IG Present’ flag could be identified based on the abnormal WBC histogram flag that is caused by reduced neutrophils, and elevated immature granulocytes and monocytes. Many of these samples have a T2 distribution flag which make the neutrophil values unreportable, and we believe this is due to the presence of immature granulocytes. To identify additional samples that have not been flagged by the XQ‐320, we performed an area under the curve analysis and found that NEUT# >7.1 × 10^9^/L or MXD% ≤ 10.6% could identify samples with ‘IG Present’ with a 71.4% and 76.8% sensitivity, respectively. This approach, besides shedding light in the common issue of unreportable neutrophil counts, could further increase the usability of XQ‐320 in different scenarios and laboratory settings and reliably send more samples directly for follow‐up examinations, without the need for a second analysis on a high‐end analyser.

To our knowledge, this is the first evaluation study of the Sysmex XQ‐320 haematology analyser, and includes comprehensive analytical performance data, extensive method comparison with the previous generation 3PD analyser of Sysmex in a cohort enriched with abnormal samples, and flagging interpretation. The study comes with some limitations. Analytical performance was conducted mainly with protocols adapted from the ICSH guidelines. One deviation was the use of two samples (representing a negative and positive as identified by the analyser) for repeatability instead of three. The CLSI EP15‐A3 guidelines were considered only for the within‐laboratory precision, where the duration of the study was 5 days instead of 20. The linearity for WBC was only possible to be assessed up to 25 × 10^9^/L, leaving a gap for strongly leucocytic samples. Another limitation was the lack of medical diagnoses or confirmation for the presence of abnormal cells with microscopic evaluation. Such information could be utilised in subsequent studies to evaluate the impact of XQ‐320 in specialised hospital wards, such as oncology.

In conclusion, the XQ‐320 showed excellent analytical performance, excellent correlation with XN‐9000 with improved performance from its predecessor in key areas, such as measuring WBC in the leucocytopenic range, and delivering precise PLT values. The XN‐like flagging system of the XQ‐320 provides more information at a glance, and in combination with parameter values can be used to identify a higher number of abnormal samples.

## FUNDING INFORMATION

The Sysmex XQ‐320 and Sysmex XP‐300 haematology analysers and all respective consumables and reagents were provided by Sysmex Swiss AG..

## CONFLICT OF INTEREST STATEMENT

KM is a full‐time employee of Sysmex Europe SE. The study was financially supported by Sysmex Swiss AG.

## Supporting information


Figure S1.

Figure S2.

Table S1.

## Data Availability

The data that support the findings for this study are available from the corresponding author upon reasonable request.
