# Supplementary material for: Evaluation of the Sysmex XQ‐320 three‐part differential haematology analyser and its flagging capabilities
Source: J Clin Lab Anal. 2024 Feb 23;38(4):e25017. doi: 10.1002/jcla.25017 (PMC10943257; doi:10.1002/jcla.25017)
Supplement: Supplementary file 1 — Figure S1. [file JCLA-38-e25017-s002.pdf]

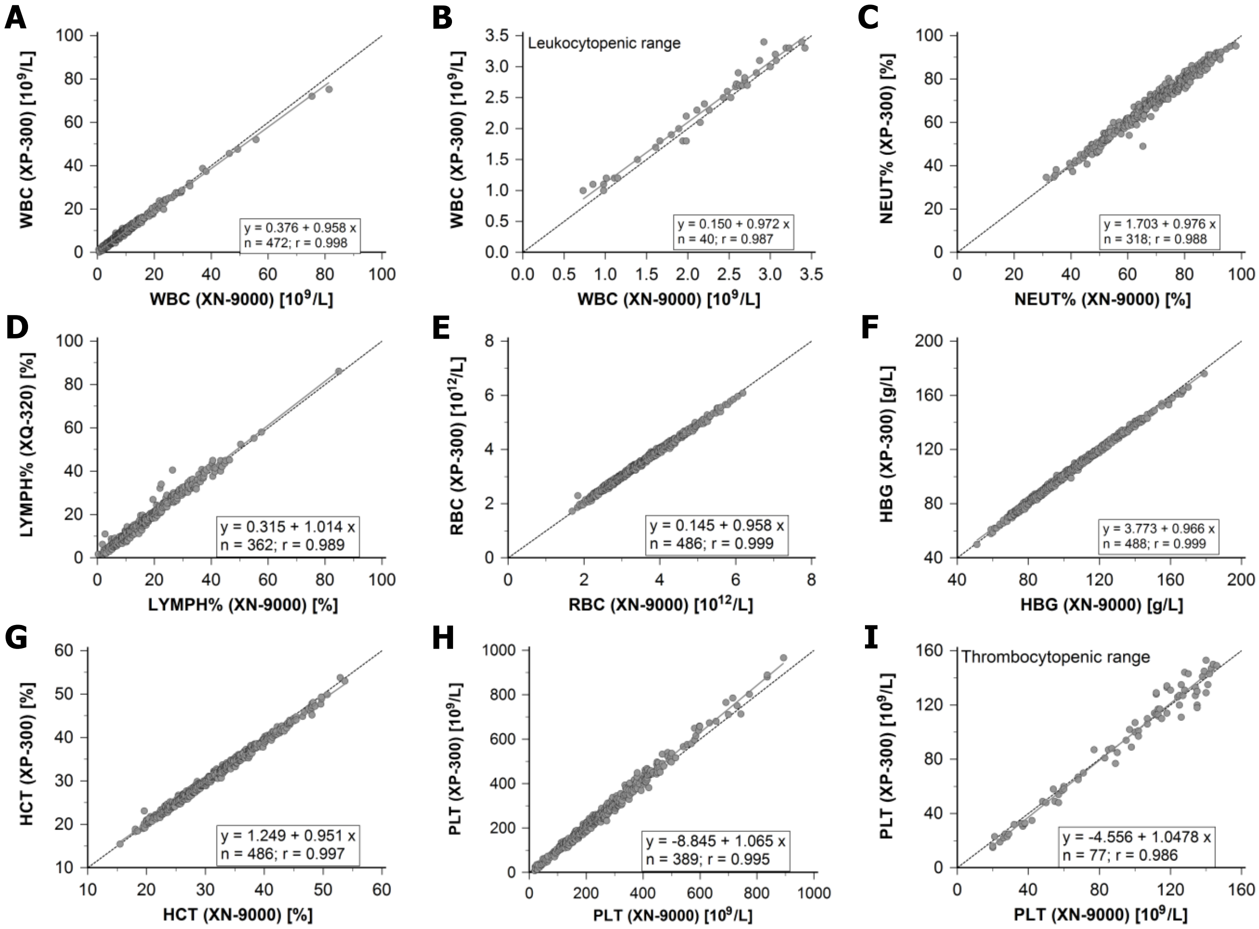

Figure S1. Correlation of selected parameters between XP-300 and XN-9000

Blood samples (n=493) were measured on the Sysmex XP-300 and Sysmex XN-9000 analysers. Correlation graphs with regression line (solid), lines of equality (dashed), regression equation and correlation coefficient (r) are shown for white blood cells (WBC) (A), WBC in the leucocytopenic range (<$150x10^9$/L) (B), neutrophil percent (NEUT%) (C), lymphocyte percent (LYMPH%) (D), red blood cell (RBC) (E), haemoglobin concentration (HBG) (F), haematocrit (HCT) (G), platelet count (PLT) (H), and PLT in the thrombocytopenic range (<$150x10^9$/L) (I).
