# Supplementary material for: Evaluation of the Sysmex XQ‐320 three‐part differential haematology analyser and its flagging capabilities
Source: J Clin Lab Anal. 2024 Feb 23;38(4):e25017. doi: 10.1002/jcla.25017 (PMC10943257; doi:10.1002/jcla.25017)
Supplement: Supplementary file 2 — Figure S2. [file JCLA-38-e25017-s003.pdf]

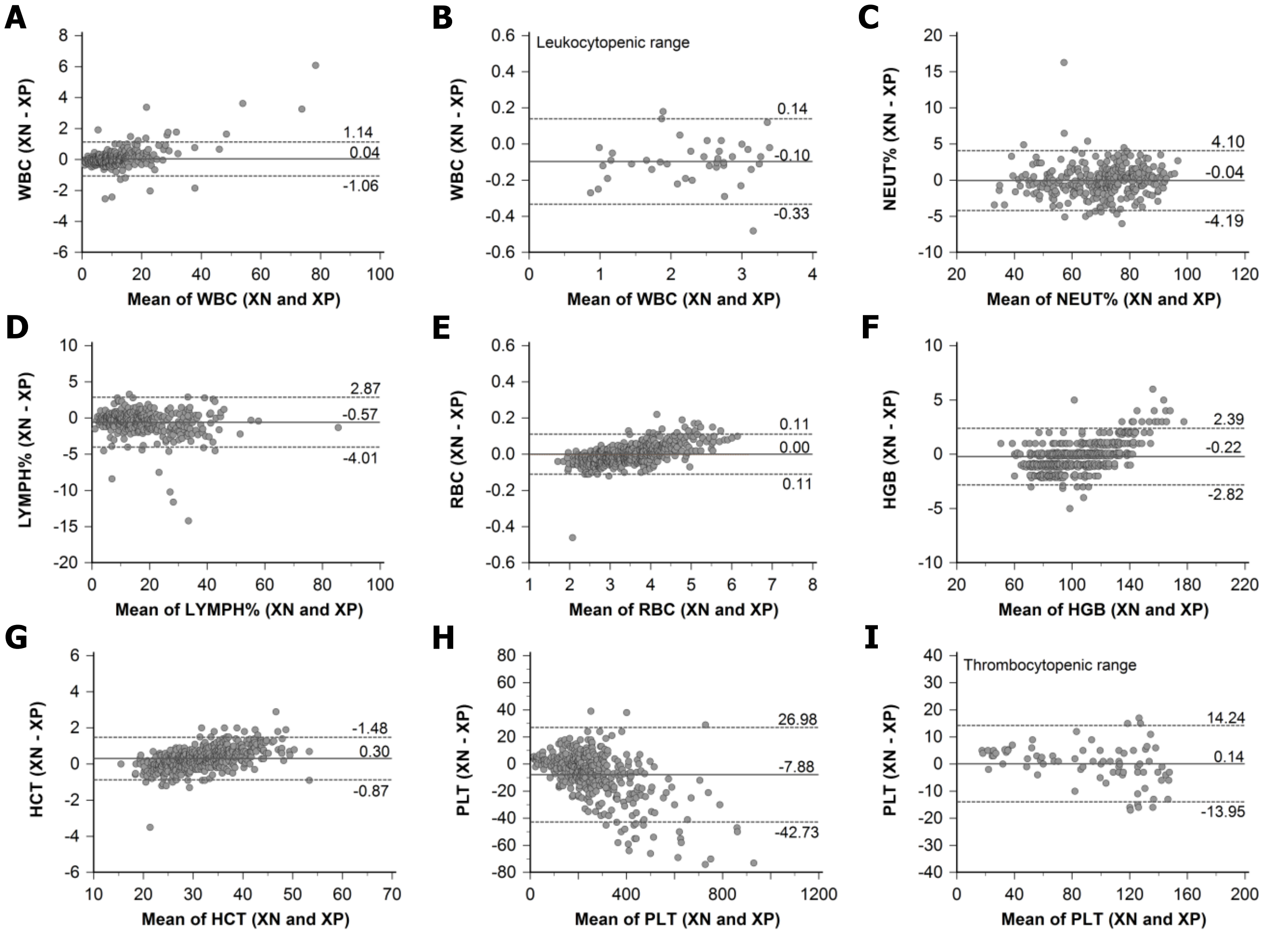

Figure S2. Bland-Altman analysis of selected parameters between XP-300 and XN-9000

Blood samples (n=493) were measured on the Sysmex XP-300 and Sysmex XN-9000 analysers. Bland-Altman plots with bias (difference of means XN – XP; solid line) and range (1.96SD; dotted lines) are shown for white blood cells (WBC) (A), WBC in the leucocytopenic range (<150x10^9/L) (B), neutrophil percent (NEUT%) (C), lymphocyte percent (LYMPH%) (D), red blood cell (RBC) (E), haemoglobin concentration (HBG) (F), haematocrit (HCT) (G), platelet count (PLT) (H), and PLT in the thrombocytopenic range (<150x10^9/L) (I).
