# Supplementary material for: Evaluation of the Sysmex XQ‐320 three‐part differential haematology analyser and its flagging capabilities
Source: J Clin Lab Anal. 2024 Feb 23;38(4):e25017. doi: 10.1002/jcla.25017 (PMC10943257; doi:10.1002/jcla.25017)
Supplement: Supplementary file 3 — Table S1. [file JCLA-38-e25017-s001.pptx]

## Slide 1
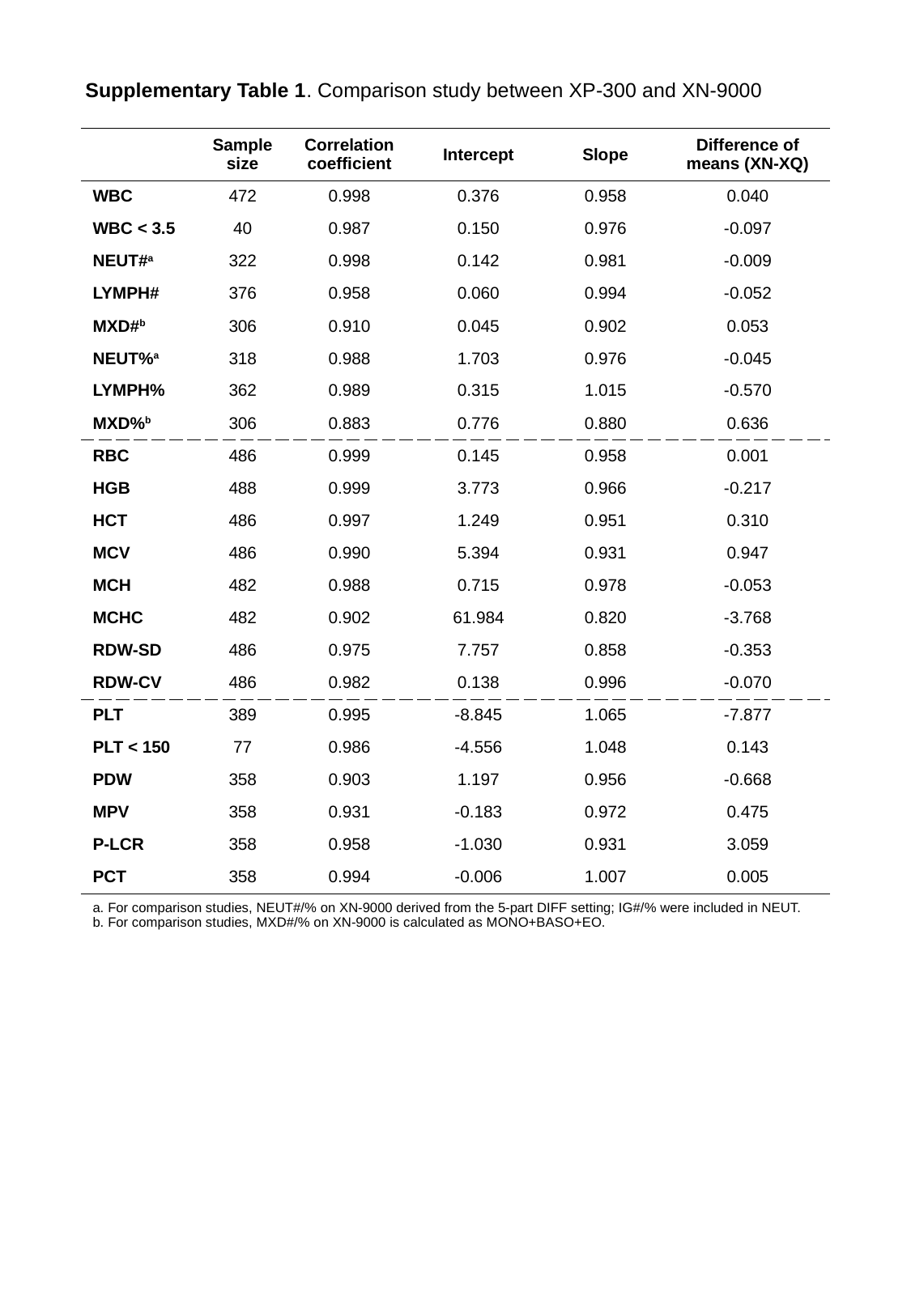

Supplementary Table 1. Comparison study between XP-300 and XN-9000
| | Sample size | Correlation coefficient | Intercept | Slope | Difference of means (XN-XQ) |
| --- | --- | --- | --- | --- | --- |
| WBC | 472 | 0.998 | 0.376 | 0.958 | 0.040 |
| WBC < 3.5 | 40 | 0.987 | 0.150 | 0.976 | -0.097 |
| NEUT#a | 322 | 0.998 | 0.142 | 0.981 | -0.009 |
| LYMPH# | 376 | 0.958 | 0.060 | 0.994 | -0.052 |
| MXD#b | 306 | 0.910 | 0.045 | 0.902 | 0.053 |
| NEUT%a | 318 | 0.988 | 1.703 | 0.976 | -0.045 |
| LYMPH% | 362 | 0.989 | 0.315 | 1.015 | -0.570 |
| MXD%b | 306 | 0.883 | 0.776 | 0.880 | 0.636 |
| RBC | 486 | 0.999 | 0.145 | 0.958 | 0.001 |
| HGB | 488 | 0.999 | 3.773 | 0.966 | -0.217 |
| HCT | 486 | 0.997 | 1.249 | 0.951 | 0.310 |
| MCV | 486 | 0.990 | 5.394 | 0.931 | 0.947 |
| MCH | 482 | 0.988 | 0.715 | 0.978 | -0.053 |
| MCHC | 482 | 0.902 | 61.984 | 0.820 | -3.768 |
| RDW-SD | 486 | 0.975 | 7.757 | 0.858 | -0.353 |
| RDW-CV | 486 | 0.982 | 0.138 | 0.996 | -0.070 |
| PLT | 389 | 0.995 | -8.845 | 1.065 | -7.877 |
| PLT < 150 | 77 | 0.986 | -4.556 | 1.048 | 0.143 |
| PDW | 358 | 0.903 | 1.197 | 0.956 | -0.668 |
| MPV | 358 | 0.931 | -0.183 | 0.972 | 0.475 |
| P-LCR | 358 | 0.958 | -1.030 | 0.931 | 3.059 |
| PCT | 358 | 0.994 | -0.006 | 1.007 | 0.005 |
| a. For comparison studies, NEUT#/% on XN-9000 derived from the 5-part DIFF setting; IG#/% were included in NEUT. b. For comparison studies, MXD#/% on XN-9000 is calculated as MONO+BASO+EO. | | | | | |
